# Histopathological lesions of congenital Zika syndrome in newborn squirrel monkeys

**DOI:** 10.1038/s41598-021-85571-1

**Published:** 2021-03-17

**Authors:** Bianca Nascimento de Alcantara, Aline Amaral Imbeloni, Darlene de Brito Simith Durans, Marialva Tereza Ferreira de Araújo, Ermelinda do Rosário Moutinho da Cruz, Carlos Alberto Marques de Carvalho, Maria Helena Rodrigues de Mendonça, Jorge Rodrigues de Sousa, Adriana Freitas Moraes, Arnaldo Jorge Martins Filho, Maria de Lourdes Gomes Lima, Orlando Pereira Amador Neto, Jannifer Oliveira Chiang, Sarah Raphaella Rocha de Azevedo Scalercio, Liliane Almeida Carneiro, Juarez Antônio Simões Quaresma, Pedro Fernando da Costa Vasconcelos, Daniele Barbosa de Almeida Medeiros

**Affiliations:** 1 Post-Graduate Programme in Virology, Evandro Chagas Institute, BR-316 Highway, km 7, Ananindeua, Pará 67030-000 Brazil; 2 Department of Arbovirology and Haemorrhagic Fevers, Evandro Chagas Institute, BR-316 Highway, km 7, Ananindeua, Pará 67030-000 Brazil; 3 Department of Pathology, Evandro Chagas Institute, BR-316 Highway, km 7, Ananindeua, Pará 67030-000 Brazil; 4 National Primate Centre, Evandro Chagas Institute, Highway BR-316, km 7, Ananindeua, Pará 67030-000 Brazil; 5 Pará State University, 2623 Perebebuí Lane, Belém, Pará 66095-662 Brazil

**Keywords:** Medical research, Experimental models of disease, Diseases, Infectious diseases, Viral infection

## Abstract

The absence of an adequate animal model for studies has limited the understanding of congenital Zika syndrome (CZS) in humans during the outbreak in America. In this study, we used squirrel monkeys (*Saimiri collinsi*), a neotropical primate (which mimics the stages of human pregnancy), as a model of Zika virus (ZIKV) infection. Seven pregnant female squirrel monkeys were experimentally infected at three different gestational stages, and we were able reproduce a broad range of clinical manifestations of ZIKV lesions observed in newborn humans. Histopathological and immunohistochemical analyses of early-infected newborns (2/4) revealed damage to various areas of the brain and ZIKV antigens in the cytoplasm of neurons and glial cells, indicative of CZS. The changes caused by ZIKV infection were intrauterine developmental delay, ventriculomegaly, simplified brain gyri, vascular impairment and neuroprogenitor cell dysfunction. Our data show that the ZIKV infection outcome in squirrel monkeys is similar to that in humans, indicating that this model can be used to help answer questions about the effect of ZIKV infection on neuroembryonic development and the morphological changes induced by CZS.

## Introduction

Zika virus (ZIKV) is a flavivirus (family *Flaviviridae*; genus *Flavivirus*) closely related to other flaviviruses that cause diseases of public health importance, such as dengue fever, Japanese encephalitis, tick-borne encephalitis, West Nile fever and yellow fever^1^^.^

ZIKV was first isolated in Uganda in 1947, and since then, the virus has been detected in Africa and later in Asia, causing outbreaks of a dengue-like febrile illness^2^. In 2007, ZIKV spread to the Pacific islands and reached South America and the Caribbean islands, likely in 2013^3,4^. In 2015, the Asian ZIKV genotype was detected in Brazil, where it was associated with congenital malformations and severe neurological disorders, such as microcephaly and Guillain-Barré Syndrome (GBS)^5–9^.

In 2016, at the beginning of the outbreak, histopathological descriptions associated with congenital Zika syndrome (CZS) in humans were scarce due to ethical issues, which limited histopathological studies^8,10–12^. Currently, several imaging studies have been reported, as well as studies that monitored the development of babies born with CZS, which has improved understanding of the syndrome caused by ZIKV^4,13,14^^.^

Many murine, swine and non-human primate (NHP) models have been tested to facilitate understanding of the pathogenesis of ZIKV infection, specifically CZS^11,15–19^. Neotropical primates belonging to the *Saimiri* genus have been used as a model for many infectious diseases, especially in studies on viral encephalitis^20–22^.

We investigated the possibility that the Asian strain of ZIKV circulating in Brazil could cause CZS-type injuries in newborn squirrel monkeys (*Saimiri collinsi*) whose mothers were infected at different stages (thirds) of pregnancy. In this work, we compare the neuroembryonic development of *S. collinsi* with that of human babies, showing that both go through critical stages of neural development in early pregnancy. Additionally, in both *S. collinsi* and human newborns with CZS, histopathological findings revealed altered migration of neuroprogenitor cells, neuronal apoptosis and necrosis of the periventricular grey matter^23–25^. Taken together, these data suggest that this species of neotropical primate is an excellent model for further study of CZS.

## Results

### Female squirrel monkeys infected with ZIKV during pregnancy

**Figure 1:**
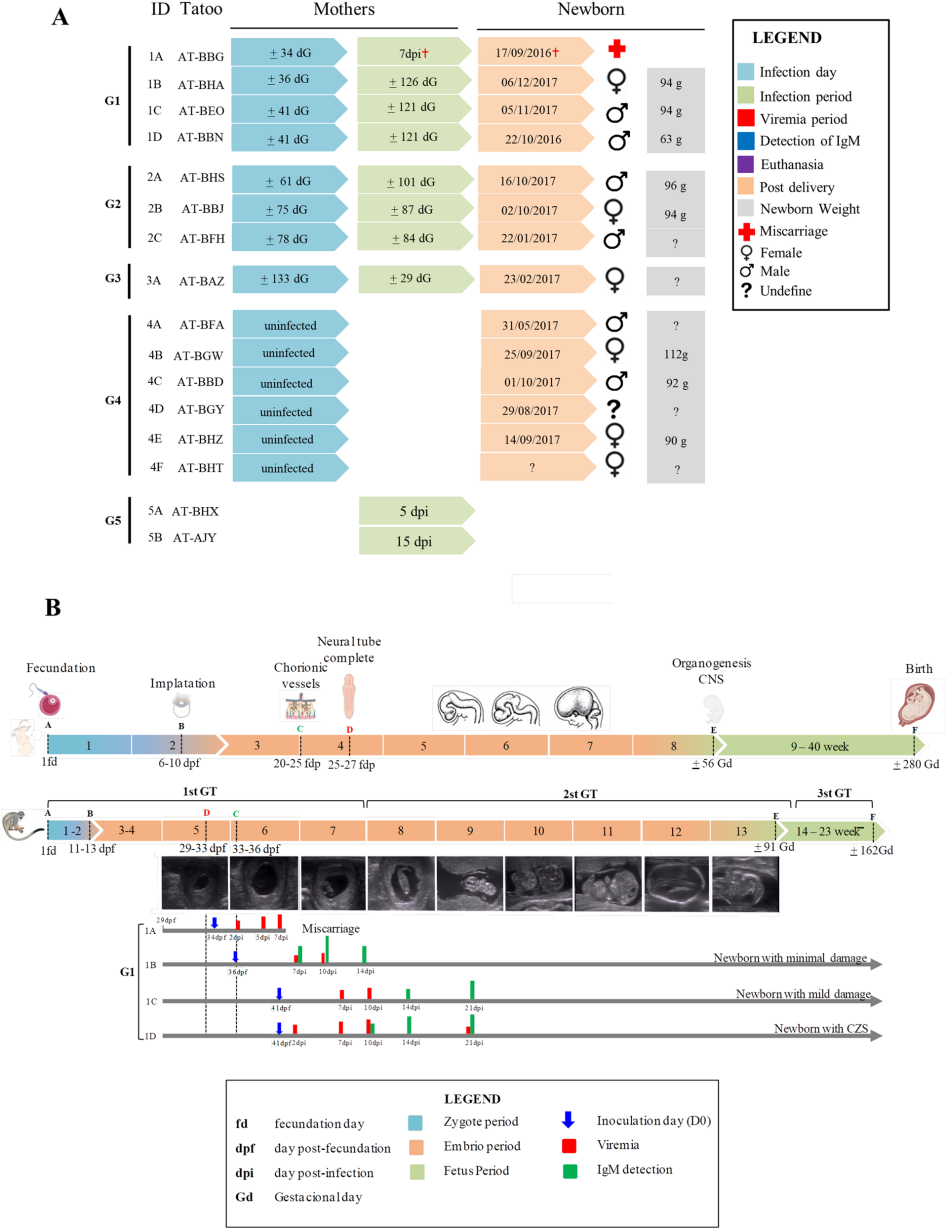
(**A**) Experimental information of squirrel monkeys (mothers and newborn) per group. (**B**) Comparison of squirrel monkey (*Saimiri collinsi*) gestational period with human gestational period from conception to birth and Time line showing embryo development from 5 to 13 Gestational weeks as well as incocultaion, viremia and IgM detection in females of Group 1.

**Figure 2:**
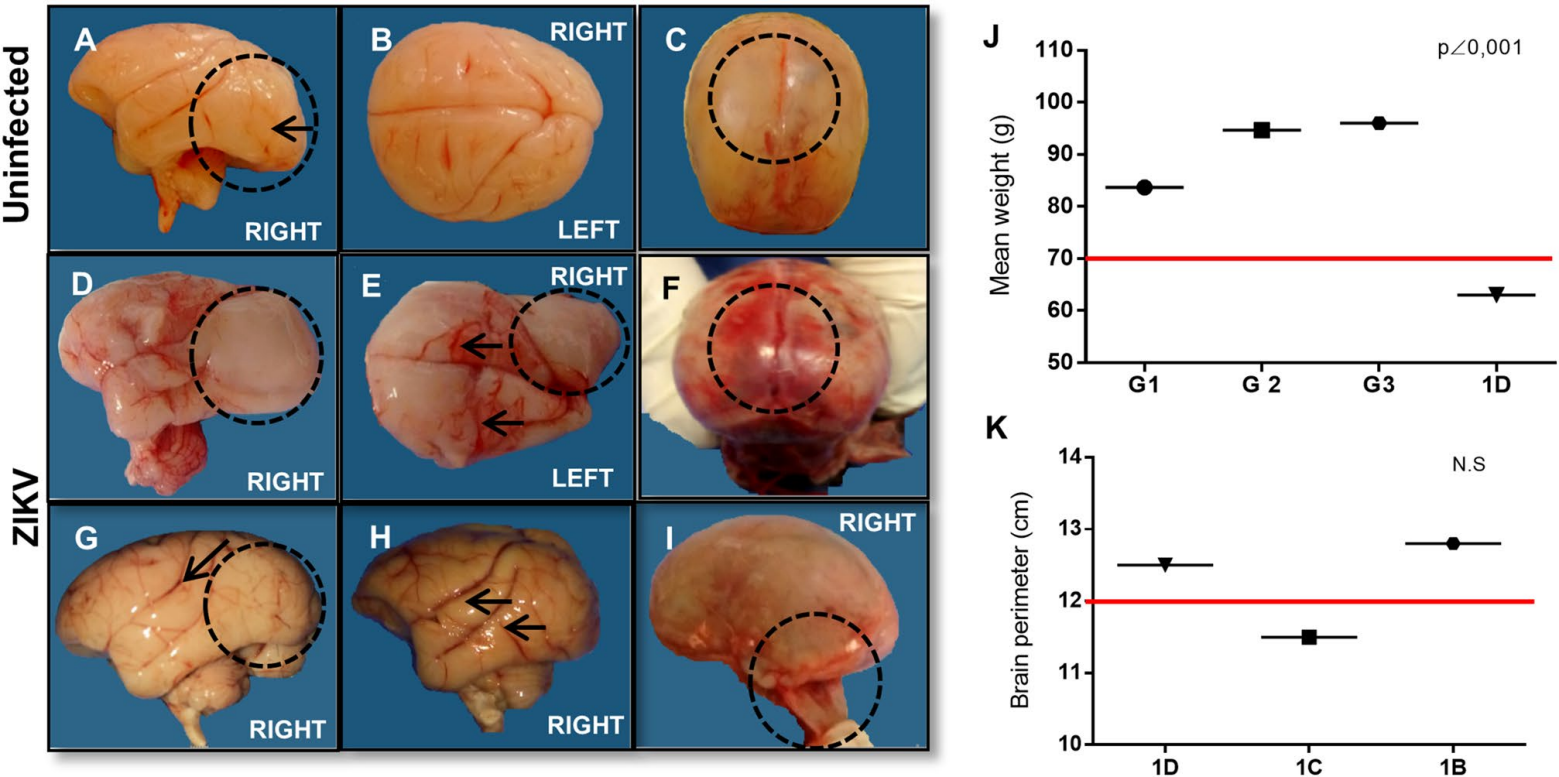
Macroscopic vascular impairment observed after autopsy and biometric differences in weight average and comparison of brain circumference. (**A**–**C**) Gross images of unfixed brain from G4 newborns (4A and 4D). (**A**) Image of right brain hemisphere of infants howing occipital gyrus (dashed black circle and black arrow). (**B**) Image of upper brain view showing normality of brain structures, presence of gyrus, and absence of vasocongestion. (**C**) The upper poster view of the skull, showing the integrity of the lambda, the point of union of the sagittal and lambdoid sutures, closing the occipital fontanelle (black arrow), absence of subarachnoid hemorrhage (dashed black circle). (**D**) Gross image of unfixed right brain hemisphere of newborn 1D (G1) showing lissencephaly, absence of occipital brain gyrus (dashed black circle), ventriculomegaly, and prolongation of the right occipital hemisphere. (**E**) Gross image of unfixed right brain of newborn 1D showing ventriculomegaly of the occipital hemisphere (dashed black circle), right side, absence of superior occipital gyrus (dashed black circle), and vasocongestion on both sides of upper view (black arrows). (**F**) Gross image of unfixed upper poster view of the skull showing Lambda malformation with no closure of the sagittal and lambdoid sutures (black arrow), and extensive subarachnoid hemorrhage (black dotted circle). (**G**) Gross image of unfixed right hemisphere of the newborn 1C (G1) brain showing lissencephaly, absence of occipital brain gyrus (dashed black circle), and vasocongestion in brain veins (black arrow). (**H**) Gross image of right brain hemisphere of newborn 1B (G1) showing vasocongestion in cerebral veins (black arrows); (**I**) crude image of the lateral view of the skull of the newborn 1D, showing lambda malformation with no closure of the sagittal and lambdoid sutures (dashed black circle; (**J**) T-test graph of means (99%) and standard deviation comparing the weight averages of the groups (G1, G2 and G3) with that of newborn 1D, the red line indicates the population mean (89.17 g); (**K**) T-test graph of means (99%) and the standard deviation comparing the brain perimeters of the newborns of group1, the red line indicates the population average (12 cm).

**Figure 3:**
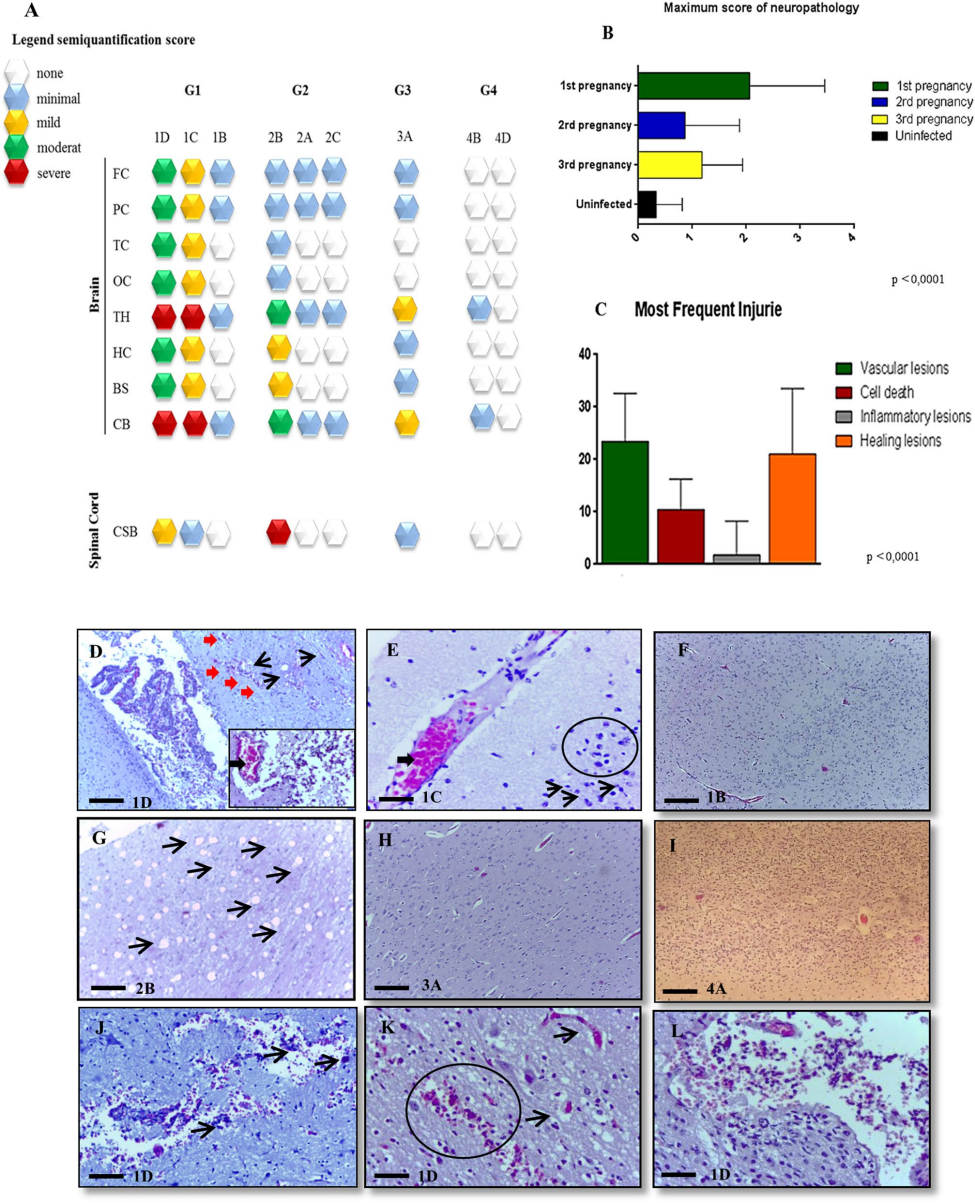
Histopathological correlations of the semiquantification score in newborns infected with ZIKV and changes found in different areas of the brain. (**A**) Summary of neuropathological lesions in the CNS of newborns infected with ZIKV (see also Table 1). The scoring system is defined in Table 1. (**B**) Maximum score for neuropathological lesions in newborns of G1, G2, G3, and G4 (uninfected newborns); statistically significant difference between groups were determined by ANOVA one way (p < 0.0001) with Tukey's post-test for multiple comparisons; red bars indicate the values that are statistically significant, G1 had ρ = 0,0003, whereas G2 and G3 presented no significant differences. (**C**) Most frequent pathological lesions found in the CNS of G1 ZIKV-infected newborns. The p values reflect ANOVA one way test (p < 0.0001), vascular injuries were the most frequent in the CNS of infected newborns, followed by scarring, neuron death, and less frequent inflammatory process. (**D**) Ventriculomegaly right side (3rd ventricle) from newborn 1D (G1), it is observed hemorrhage (red arrows), slight mononuclear inflammatory infiltrate, perivascular necrosis/apoptosis (black arrows) (magnification ×100 and scale bar 25 µm), perivascular edema, and vasocongestion (box, magnification ×400 scale bar 50 µm). (**E**) Histopathological image of 3rd ventricle from 1C newborn brain showing vascular congestion (large black arrow), perivascular necrosis/apoptosis (thin black arrows), and satellitosis (black circle (magnification ×100 and scale bar 25 µm). (**F**) Histopathological image of OC lobe 1B newborn from G1 (magnification ×100 and scale bar 25 µm), few injuries compared to the other two newborns in the same group (Fig. 2D, E), and the negative control (**I**). (**G**) Cervical spinal cord (CSC) from 2B newborn (G2) ZIKV infected showing compatible image with Wallerian degeneration or CSC malacia (magnification ×100 and scale bar 25 µm). (**H**) Histopathological image of OC lobe 3A newborn from G3 (magnification ×100 and scale bar 25 µm), few injuries compared to the other newborns in the other groups and the negative control (**I**). (**I**) Histopathological image of OC lobe 4A newborn G4, negative control, few injuries, only edema, and minimal vasocongestion (magnification ×100 and scale bar 25 µm). (**J**) OC lobe 1D newborn (magnification ×100 and scale bar 25 µm), calcification (black arrow) in the neural parenchyma. (**K**) FC lobe of 1D newborn (magnification ×100 and scale bar 25 µm), vascular lesions in the neural parenchyma. (**L**) FC lobe 1D newborn (magnification ×100 and scale bar 25 µm), are hemorrhagic meningitis.

For this study, the four ZIKV-infected groups (G1, G2, G3, and G5; Fig. 1A) were inoculated via the intradermal (i.d.) route with a dose of 1.0 × 10^5^ plaque-forming units (PFUs). Based on ultrasound (US) evaluation, embryo development and previous information about neotropical NHPs, the animals from G1 were further divided into two subgroups using a limited time point approach for chorionic vessel detection. Therefore, two animals (1A and 1B) were infected before 37 gestational days (Gd), and the other two animals (1C and 1D) were infected after 37 Gd. Of the ten infected pregnant females, seven completed gestation and gave birth to their newborns naturally. One animal (1A) had a miscarriage at 7 days post-infection (dpi), at approximately Gd 41 (Fig. 1A). Confirmation of ZIKV infection in animal 1A was shown in our previous paper^26^. Macroscopic (Fig. 2) and microscopic (Fig. 3) changes in the central nervous system (CNS) were observed in the offspring of females infected at different gestational periods and compared with those in the CNS of the newborns in the negative control group (G4) (Fig. 2A), as well as with the information available on the lesions described in humans newborns. Our experimental model mimicked the variability of lesions caused by ZIKV during embryogenesis as well as the association between viremia and the maternal–fetal immune response^26^. Figure 1B summarizes these previous findings. Viral infection in the offspring after peripheral transmission was also a testament to the ability of ZIKV to break the physical barrier of the placenta and the blood–brain barrier during the different gestational periods of squirrel monkeys.

### Macroscopic changes in the CNS

Before necropsy, the animals were weighed and measured; after necropsy, a macroscopic examination in search of morphological changes was performed, and the observations were recorded. The newborns in G1 were compared with those in G4 (uninfected/Fig. 2A–C); G1 neonates showed several changes in brain structures, including lissencephaly, a simplified gyral pattern, blood–brain barrier damage (Fig. 2D,G), edema and vasocongestion (1C, Fig. 2H), with multifocal areas of subarachnoid haemorrhage (1D, Fig. 2F). Newborn 1D had right lateral ventriculomegaly (Fig. 2E) in the region of the third ventricle, and the occipital fontanelle was open and not fully formed (Fig. 2I), indicating a delay in development. Newborn 1B showed a few changes, such as vasocongestion and edema in the parenchyma. Macroscopic vascular changes, such as edema, congestion and haemorrhage, were also observed in newborns in G2, although the changes were more discrete than those observed in G1. The newborns in G3 did not show any macroscopic changes. The integrity of the cerebellum (CB), brain stem (BS) and cervical spinal cord was observed in all newborns from G2 and G3. The average weight of the newborns in G1 was 83.67 g, whereas newborn 1D weighed only 68 g. The average weights of newborns in G2 and G3 were 94 g, 67 g and 96 g, respectively (Fig. 2K). The brain circumferences of the G1 offspring were 12.8 cm (1B), 11.5 cm (1C) and 13.5 cm (1D) (Fig. 2K). The most remarkable finding was the ventriculomegaly seen in newborn 1D, which explains the projection of the posterior region of the brain.

### Microscopic analysis of the CNS

Semi-quantitative analysis of the histological sections of the CNS of newborns from all infected groups showed histopathological alterations, usually to a severe degree (Fig. 3A,B). Among the histopathological lesions, the most frequent were vascular changes, cell death, vascular and perivascular edema, and inflamed and healed lesions (Fig. 3C). Analysis of variance (p < 0.0001) of these lesions indicated the prevalence of vascular lesions in the cerebral epithelium of newborns from all groups analysed (G1–G3), with extensive vascular proliferation, vasocongestion, pericellular and vascular edema and cell death. In G1, newborns 1C and 1D presented lesions with greater severity and more abundance in the evaluated brain areas. Figure 3B shows how the lesions were distributed among the infected groups. The brain of newborn 1D had increased endothelial haemorrhagic changes (Fig. 3D,J,L), neuronal death, perivascular edema, gliosis and satellitosis in the frontal cortex (FC) (Fig. 3D); vascular proliferation in the temporal cortex (TC); and gliosis and haemorrhagic lesions in the hypothalamus (HT) (Fig. 3E). In the areas of neuronal death in the occipital cortex (OC), CB and BS, cell death was frequent. Newborn 1C had lesions in several analysed areas of the CNS that ranged from medium to severe (Fig. 3B), with the most severe injuries in the OC, CB and BS, where there was severe and abundant necrosis. Interestingly, newborn 1B had minimal lesions in the focal areas, characterized by endothelial reactivity, small foci of edema, and red blood cells spilling out of the FC (Fig. 3F). The newborns in G2 also showed lesions, although to milder degrees than those seen in the newborns in G1. Newborn 2B exhibited the greatest effects, with myelin degeneration similar to that seen in severe Wallerian degeneration (Fig. 3G). The other animals had a minimal degree of injury (Fig. 3A). The only newborn in G3 showed minimal lesions (Fig. 3H) in the OC, CB and BS, with low endothelial reactivity and a few areas of edema and congestion. In newborns from G4 (Fig. 3I), few injuries (only edema and minimal vasocongestion) were observed.

### ZIKV detection in fetal tissues

**Figure 4:**
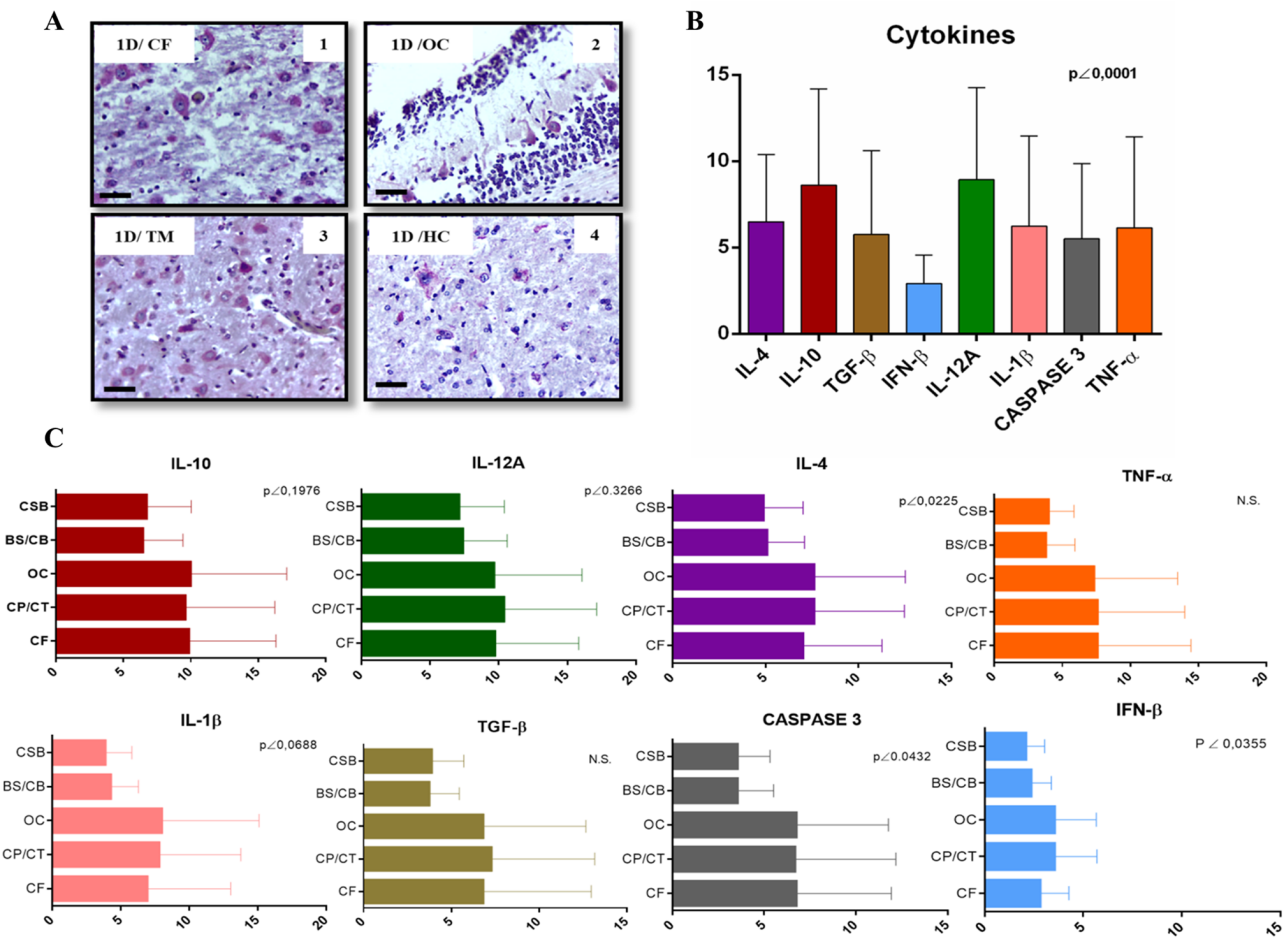
Immunohistochemistry (IHC) analysis of tissue brain from G1 newborns infected with ZIKV. (**A**) IHC markings of ZIKV-positive antigen/antibody reaction. In the box, the highlighted shows cytoplasmic immunostaining in CT neurons, degenerated neurons marked on the right side of the image, and glial cells marked on the lower left side. ×400 magnification; scale bar, 50 µm. (**B**) ANOVA one-way analysis with Kruskal–Wallis statistical post-test for multiple comparisons (p < 0.0001) of the cytokine panel tested on G1 newborns; color bars reflect the quantification of cytokine markings in sections from CF, CP/CT, OC, BS/CB, and CSC of the brain analyzed. (**C**) ANOVA one-way analysis showing quantification of cytokines at the different areas of the brain.

Using RT-qPCR, high ZIKV loads were detected in fetal brain tissues from newborn 1D (1.48 × 10^1^ RNA copies/mg of tissue), while the virus genome was undetectable in the other newborns (using a standard curve analysis, the estimated limit of detection for the RT-qPCR assay was ~ 25 RNA copies/mg of tissue). However, the presence of antigens against ZIKV was confirmed in all newborns by immunohistochemistry (IHC). The areas of the brain with the highest amount of immunostaining are shown in Fig. 4B, highlighting neurons and glial cells in the CB/BS, FC and OC, where substantial immunostaining was observed in areas with a high number of motor neurons close to vascular lesions. In most newborns, no significant inflammatory infiltrate was observed; however, in G1 animals (1C and 1D), which had the highest degree of lesions; an extensive perivascular lymphomononuclear infiltrate was observed close to the affected lateral ventricle.

### Immune response against ZIKV in CNS tissues

Analysis of the cytokine and inflammatory marker profile in CNS tissue samples from newborns in G1, who had the highest number of lesions, showed high IL-10 (p < 0.19) and IL-12A (p < 0.0001) expression; followed by IL-4 (p < 0.0001), with a positive correlation (ρ = 1) in infant CNS tissue; and IL-1β (p < 0.068), TGF-β (N.S.) and TNF-α (N.S.); low IFN-β expression (p < 0.0355) was detected. We observed a predominance of Th2 markers over Th1 markers, such as IL-10 and IL-4, which act in tissue reconstruction and modulation of the expression of other cytokines, such as TNF-α, accompanied by the pro-inflammatory cytokines IL12-A, TNF-α, IL-1β and IFN-β (Fig. 3B). Immunoexpression of cytokines, especially IL-4, IL-10 and IL-12A, occurred in the brain parenchyma. The expression was mainly observed in areas containing neurons, in addition to areas with dead neurons and low mononuclear inflammatory infiltrate. In particular, an increase in Caspase 3 expression (p < 0.04) was observed in the parenchyma, especially in areas with intense neuronal damage to the cortex, where intense staining was observed in vacuolated and apoptotic neurons (Fig. 4A). Cytokine expression levels in specific brain areas are shown in Fig. 4C.

## Discussion

This study showed that experimental infection of *S. collinsi* females in different gestational thirds (GT) with the Asian genotype of ZIKV produced results similar to those of infection in humans, demonstrating the epidemiological pattern of CZS occurrence in susceptible newborns. CNS disorders were mainly observed in newborns from females infected in the first period of pregnancy, similar to findings in babies with CZS^3,8,11,22,33^.

The first trimester of pregnancy in humans and the corresponding periods in these primates (1st GT and 2nd GT) are fundamental for proper development of the main organ systems (organogenesis), especially the brain and its associated neuroprogenitor cells^34^. Comparatively, the gestation time of NHPs is approximately 162 days, with the 1st GT of *S. collinsi* lasting approximately 54 days (Fig. 1B), while in humans, gestation lasts approximately 280 days. Relevantly, in this study, we were able to mimic severe lesions in newborns when maternal infection occurred after detection of chorionic vessel formation (Fig. 1B), thus demonstrating that this stage of embryonic development is an early period of organogenesis equally crucial in squirrel monkeys and humans.

Two newborns (1C and 1D) in G1, whose mothers were infected at 41 Gd, coinciding with the period after separation of fetal circulation from maternal circulation, exhibited the most serious injuries (Fig. 1B). On the other hand, two situations occurred for animals infected before complete formation of the chorionic vessel: miscarriage (1A, infected at 34 Gd) and minor injury (1B, infected at 36 Gd). The balance between virus replication and both the intensity and speed of humoral response establishment could be a determining factor for irreversible tissue damage or virus clearance.

Combining the findings reported by Imbeloni et al.^26^ with the results of this study, in animal 1A, the virus began to be detected at 2 dpi, and the highest titre occurred at 7 dpi, the same time as spontaneous abortion and without any IgM detection. For animal 1B, anticipated and higher maternal IgM titres were associated with late viremia and below levels seen in the other females in this group, which could have been crucial in protecting the fetus from ZIKV infection and minor brain damage.

Robust maternal adaptive immune responses as well as the placenta form a protective barrier for the fetus in early pregnancy. However, once the formation of the placental barrier is complete, maternal immunity can be restricted, and the main defences against infection are driven by the immature immune system of the developing fetus^5,35^. Consequently, the chance of mild or severe brain damage development in the fetal brain increases, such as in newborns 1C and 1D. Furthermore, the maternal response likely contributes to the lesions in the fetus. In animal 1D, the higher and prolonged maternal viremia associated with lower and delayed IgM detection and persistent viral infection in the fetus until delivery resulted in severe damage to the fetal brain, similar to CZS. Previous studies have reported that persistent viral infection in maternal tissues can provide a reservoir that facilitates vertical transmission of ZIKV, which provides a basis for understanding the wide range of fetal developmental abnormalities caused by ZIKV^36,37^.

The frequency of CZS-like lesions in our study (67%, 2/3) was close to that found in females of other NHP species infected early^8,11^. Some studies have been able to reproduce ZIKV infection in NHPs, including rhesus, cynomolgus and even neotropical primates, such as marmosets and different species of squirrel monkeys. However, lesions consistent with CZS have not been observed in such studies^22,37^.

Biometric differences, such as differences in weight and brain circumference, were evident among newborns in G1 (Fig. 2J,K). These data suggest that in newborn 1D ZIKV infection led to an increase in brain circumference, with an increase in the occipital region due to ventriculomegaly (Fig. 2E,F), and general growth restriction, although we did not observe gross microcephaly. As organogenesis in squirrel monkeys is nearly complete at the end of the 1st GT, the malformations consistent with CZS identified in this newborn (1D) did not occur in newborns from other infected groups in the later stages of pregnancy which often occur in human babies^24,36^.

Macroscopic examination of the brains of G1 newborns showed severe vascular lesions, such as vasocongestion, subarachnoid haemorrhage and edema (Fig. 2E–H), which were similar to brain lesions caused by other encephalitic flaviviruses^38–40^. In newborn 1D, the right occipital lobe in the region of the 3^rd^ and lateral ventricles was enlarged (Fig. 2E). Lissencephaly and reduced cerebral gyri in the occipital region of the right hemisphere were seen in newborns 1D and 1C (Fig. 2D,G). Newborn 1D presented lambda malformation without closure of the sagittal and lambdoid sutures (Fig. 2I). Lesions such as lissencephaly, reduced cerebral gyri in the occipital region and ventriculomegaly have been previously described in rhesus monkeys by Martinot et al.^8^ and Adams Waldorf et al.^11^. In human children exposed to ZIKV during pregnancy, magnetic resonance imaging (MRI) revealed ventriculomegaly, simplified gyral pattern and lissencephaly^23,40–42^.

The histopathological lesions found in the CNS, including cerebral microcalcification (which was observed only in newborn 1D, Fig. 3J—14.28%, 1/7) and cerebral parenchymal (Fig. 3D,E,G,K) and meningeal haemorrhages (Fig. 3L), have already been described in humans and other species of NHPs^43–45^. Of the infected newborns in our study, only one (14.28%, 1/7) presented ventriculomegaly (Fig. 2E), while two (28.57%, 2/7) presented lissencephaly and an absence of occipital cerebral gyri (Fig. 2D,G). ZIKV antigen immunostaining was observed in the cytoplasm of neurons and necrotic and degenerating glial cells, as shown in Fig. 4 (1–4)^8,36,37,46^. Cumberworth et al.^28^ examined the ZIKV envelope protein via IHC and found that all major types of CNS neural cells were labelled.

The results of our macroscopic and histopathological analyses, integrated with the immunohistochemistry and immunoexpression data and the evidence of viral expression in the tissues, demonstrated the possibility of neurological persistence of ZIKV infection in the CNS until birth, as indicated by the expression of pro-inflammatory mediators^33,34^. We detected low IFN-β expression, mainly in the areas of the OC, PC/TC and HR, in the nervous tissues of newborns in G1 (Fig. 4B,C), likely related to development of a classic antiviral response (Th1) against ZIKV, which is crucial for inhibiting viral replication^36–39^. Based on previous studies, ZIKV appears to selectively inhibit translation of IFN-I mRNA, while translation of other host viral proteins remains intact. Blocking IFN-I translation appears to be an exclusive feature of ZIKV because other viruses do not block translation or secretion of this cytokine^50^.

In animals, low IFN-I and IFN-II levels promote ZIKV replication and spread of ZIKV infection to the CNS^51^. We believe that the low expression of this cytokine in the nervous tissue of newborns indicates the specific nature of the immune avoidance mechanism that likely contributes to the viability of ZIKV until the birth of the fetus. Although replication inhibition mechanisms become active to a small extent, ZIKV remains in the escape sites of the CNS^33,36,37^.

The production of different cytokines has a strong influence on the infection environment and modifies the immune response mechanisms. The Th1 cell profile mainly produces IFN-γ, IL-12 and TNF-α, and malfunction of this response has been correlated with severe viral infections^41^. Our data demonstrated an increase in IL-12A and IL-4 expression. The balance between IL-12, favouring Th1 responses, and IL-4, favouring Th2 responses, promotes a balanced immune response in situ^42^. An increase in IFN expression is supported by the presence of IL-12A, a cytokine that classically induces IFN-γ production, which in turn can modulate microglial activity in response to the presence of ZIKV^11,46,52^.

Sousa et al.^47^ and Azevedo et al.^46^ linked the Th1 lymphocyte response to positive immunostaining for IFN-γ, IFN-α, IFN-β, IL-6, IL-12A, IL-1β and TNF-a. Our panel showed positive IFN-β, IL-12A, IL-1β and TNF-α immunostaining in the tissues of squirrel monkeys, demonstrating a similar profile in the nervous tissues of the newborns in G1, who were born after maternal infection. The increase in the level of pro-inflammatory cytokines suggests a coordinated protein response that may be associated with induction of cellular damage mechanisms. In fact, cytokines are the main mediators that trigger neurotoxicity and the production of reactive oxygen and nitrogen intermediates directly involved in cell death. In addition, IL-1β and TNF-α may also be involved in the apoptotic cell death mechanism because they are associated with activation of death receptors and inflammasome triggers, respectively^49,53,54^.

Positive immunostaining for IL-4, IL-10, IL-33, IL-37 and TGF-1β, indicative of the presence of a Th2 response, has already been reported in other viral encephalitis cases and may be related to simultaneous development of suppressive agents and an antiviral response, which are necessary for elimination of the infectious agent without causing major inflammatory damage^46,47,49,54,55^. The involvement of the Th2 profile indicates cell death via necrosis and apoptosis, and the induction of the M2 microglial response indicates that involvement of the Th2 profile is closely related to the pathogenesis of ZIKV. The Th2 profile is related to cell death, giving rise to a more favorable environment for viral replication and activating M2 macrophages^11,46^.

IL-4 and IL-10 immunoexpression was increased in the nervous tissue of G1 newborns, in which necrosis of neurons in the cortical layer of the OC and BS/CB was much more intense. Apoptosis is likely the main type of cell death involved in the development of CNS damage induced by ZIKV. We observed increased Caspase 3 expression in the parenchyma, including in areas of the cerebral cortex, with a prevalence of immunostaining in the OC, PC/TC and FC (Fig. 4B,C). This finding is consistent with results from in vitro studies suggesting that apoptosis is the main factor in neuronal cell death and reduced brain mass in humans^55,56^.

Our data also demonstrate that the in situ immune response against ZIKV in the CNS of newborns of this species is complex; the expression of Th2 and Th1 cytokines is predominant, but other cytokine profiles are involved to a lesser extent. Mechanisms of neuronal cell death include necrosis and apoptosis, which are induced by different immunological factors and likely by direct action of the virus itself, which has already been reported in humans^46,47^. However, studies in experimental models elucidate the pathogenic mechanisms and immunological factors involved in the host response against ZIKV infection in the CNS.

Although our findings help clarify some aspects of CZS, a better understanding of the role of placental permissiveness is still necessary and would improve understanding of the mechanisms involved in the different tissue responses observed in newborns in G1, whose mothers were infected during the first GT. However, despite the need for further studies in this animal model, our data suggest strong vascular involvement and dysfunction of neuroprogenitor cells during fetal development, with involvement of pathological mechanisms that occur early in pregnancy. Thus, in addition to assisting in chronological comparison of the neuroembryonic development of *S. collinsi* with that of humans, these findings corroborate what has already been observed in other species of NHPs and increase knowledge of the pathogenesis of CZS.

## Methods

### ZIKV strain BeH815744

The isolate was provided by the Section for Arbovirology and Haemorrhagic Fevers (SAARB) of the IEC. The ZIKV BeH815744 strain (GenBank KU365780) was isolated, sequenced and genetically characterized as previously described^4^. The viral isolate was amplified and titrated using the Vero cell line; cells were grown in 199 medium supplemented with 2% fetal bovine serum (FBS) and antibiotics^10^. Females were infected via the intradermal (i.d.) route with 1.0 × 10^5^ PFUs of virus inoculum, which was injected into the right intercostal space above the nipple.

### Study design

Ten female squirrel monkeys (*S. collinsi*) from a colony in the CENP were naturally mated. Pregnancy was confirmed by US, and for the purposes of this study the gestational time was considered to be approximately 162 days, as previously published^26^. According to the GT at the time point of i.d. ZIKV inoculation, the females were divided into three groups: G1 (n = 4), G2 (n = 3) and G3 (n = 1). One group (G4) with two non-infected pregnant monkeys was used as a control, and their newborns were sacrificed (4A and 4B) for histopathological comparisons (Figs. 1A, 2B).

### Necropsy of animals

The necropsy of the mothers and their newborns was performed 4 days after delivery. Macroscopic examination of the CNS was performed and noted on a specific form. Samples from each area of the brain, as well as from the cervical spinal cord, were fixed in 10% buffered formaldehyde and processed after 48 h.

### Histological analysis of CNS tissues

The assessment of the CNS was performed as described by Mlakar et al.^14^. Sections (5 μm) of different anatomical regions of the CNS, the frontal (FC), parietal (PC), temporal (CT) and occipital (OC) cortices, and the thalamus (TH), hippocampus (HC), brain stem (BS), cerebellum (CB) and cervical spinal cord, were included in paraffin blocks and stained with hematoxylin and eosin (H&E). The sections were hydrated in a decreasing alcohol gradient (100%, 80%, 70% and 50% ethyl alcohol), washed, stained with hematoxylin dye, washed again and stained with eosin dye, then washed, sequentially dehydrated in an increasing sequence of alcohol (ethyl alcohol 50%, 70%, 80% and 2 repetitions at 100% concentration) and finally two fixations for the conservation of the material (xylol I and II).The stained sections were analysed by optical microscopy (Zeiss, Oberkochen, Germany), with magnifications of 100× and 400×, by two independent pathologists, and the slides were viewed proximal to the meninges, parenchyma and perivascular region. Quantitative analysis was performed (Table 1)^8^ using a semi-quantitative scoring system (0–4) based on the increasing degree of severity of pathological lesions observed in the CNS of the newborns.

**Table 1:** Semiquantitative scoring system for histopathological lesions of the newborns SNC used throughout the study.

Neuropathology, Apoptosis, and Neuroprogenitor Scoring Systems in newborns of squirrel monkeys			
Score	Severity	Description	Examples/interpretation
0	None	no lesions observed	N/A
1	Minimal	Focal, small lesion observed, Not observed in control animals but significantly questionable, could still be within normal limits or back ground injury	Single focus, rare lymphocytes, small area of extravasated red blood cells outside of vessels (focal microhemorrhage)
2	Mild	Multifocal small lesions in a single brain section	Endothelial reactivity, perivascular necrosis, large foci of extravasated red blood cells outside a vessel (hemorrhage)
3	Moderate	Multifocal larger lesions in a single brain section	Large aggregate of persistent neuroprogenitor cells in unusual patterns in the brain with or without vascular changes/vasculitis in brain or spinal cord
4	Severe	Multifocal larger lesions in a single brain section with similar lesions in other brain sections; or fetal loss associated with tissue viremia	Necrosis/apoptosis associated with vascular change/vasculitis, increased numbers of neuroprogenitor cells (gliosis, satellitosis), ‘red-dead’ neurons, congestion, and neuron clustering

### Immunohistochemistry

The streptavidin–alkaline phosphatase method was adapted to detect the viral antigen using a polyclonal anti-ZIKV antibody produced in mice at the IEC. Based on previous studies^27,28,57^, the peroxidase method was used for tissue immunostaining with specific monoclonal antibodies to track cytokines and markers of apoptotic cell death pathways to evaluate a small panel designed for this study and containing the following markers: IL-10, TGF-β, IFN-β, IL-12A, IL-1β, Caspase 3 and TNF-α (see Supplementary Data 2 for specifications and dilutions)^29^.

### Detection and quantification of the ZIKV genome

Infected tissues were mixed with PBS (pH 7.4) on Tissuelyser (Qiagen, Carlsbad, USA) and subjected to viral RNA extraction using a TRIzol Plus RNA Purification kit (Thermo Fisher Scientific, Carlsbad, USA). The RT-qPCR protocol^16^ was adapted to use QuantiTect Probe PCR kits (Qiagen, Carlsbad, USA), and RT-qPCR was performed on a 7500 Real-Time Fast PCR system (Thermo Fisher Scientific) with the standard curve and synthetic amplification target included in the PUC plasmid for RNA quantification, following the methods described by Nunes et al.^31^. The RNAase P gene was used as an internal endogenous control^32^. The limit of detection was ~ 25 RNA copies/mg of tissue, as reported by Lanciotti et al.^30^. Data on viremia (RT-qPCR) and the immune response (ELISA IgM) of mothers are available in Supplementary Data 1.

### Statistical analyses

For univariate analysis, frequencies and measures of central location and dispersion were obtained. The hypotheses were tested using one-way ANOVA with a Kruskal–Wallis test. The level of significance adopted was α = 0.01 (p ≥ 0.01, not statistically significant (N.S.); p < 0.01, significant; p < 0.001, very significant; and p < 0.0001, extremely significant). We used Pearson's correlation (ρ), assuming only values between − 1 and 1, to indicate positive or negative correlations. All analyses were performed with Prism 6.0 software (GraphPad, San Diego, USA).

### Ethical statement

The experimental infection occurred according to the rules of the Chico Mendes Institute for Biodiversity Conservation (ICMBio-No.: 53391-1). The study was carried out at the National Primate Centre (CENP) and was approved by the Ethics Committee on the Use of Animals of the Evandro Chagas Institute (CEUA/IEC-no. 010/2016) in accordance with the provisions of Brazilian Law No. 11,794 (October 8, 2008), Brazilian Decree No. 6,899 (July 15, 2009) and with the rules issued by the National Council of Animal Experimentation Control (CONCEA).

## Supplementary Information

Supplementary Information 1.

Supplementary Information 2.
