# Supplementary figures and images for: Histopathological lesions of congenital Zika syndrome in newborn squirrel monkeys
Source: Sci Rep. 2021 Mar 17;11:6099. doi: 10.1038/s41598-021-85571-1 (PMC7971060; doi:10.1038/s41598-021-85571-1)

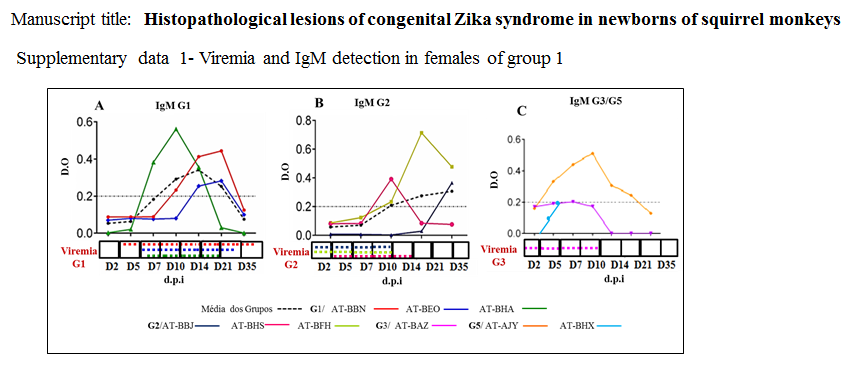

Supplement: Supplementary file 1 — Supplementary Information 1. [file 41598_2021_85571_MOESM1_ESM.tif]

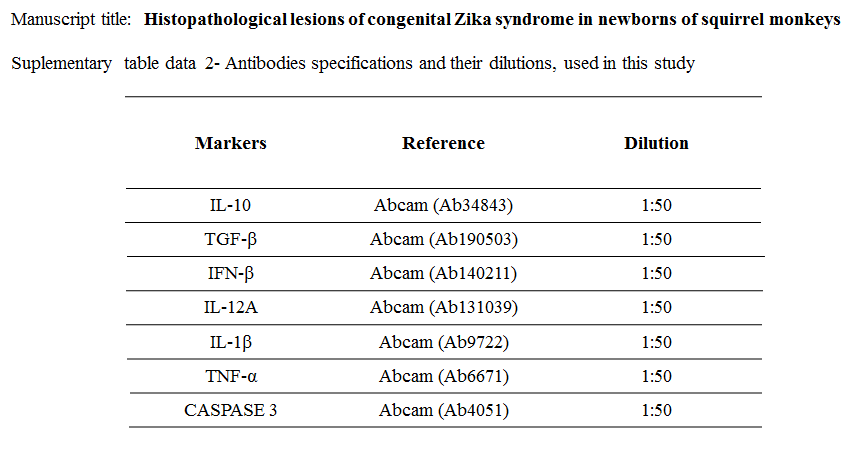

Supplement: Supplementary file 2 — Supplementary Information 2. [file 41598_2021_85571_MOESM2_ESM.tif]
